# A Genome-Wide Screen of Deletion Mutants in the Filamentous Saccharomyces cerevisiae Background Identifies Ergosterol as a Direct Trigger of Macrophage Pyroptosis

**DOI:** 10.1128/mBio.01204-18

**Published:** 2018-07-31

**Authors:** Kristy Koselny, Nebibe Mutlu, Annabel Y. Minard, Anuj Kumar, Damian J. Krysan, Melanie Wellington

**Affiliations:** a Department of Pediatrics, University of Rochester Medical Center, Rochester, New York, USA; b Department of Molecular, Cellular and Developmental Biology, University of Michigan, Ann Arbor, Michigan, USA; c Department of Molecular Physiology and Biophysics, Carver College of Medicine, University of Iowa, Iowa City, Iowa, USA; d Department of Microbiology and Immunology, University of Rochester Medical Center, Rochester, New York, USA; e Department of Pediatrics, Carver College of Medicine, University of Iowa, Iowa City, Iowa, USA; f Department of Microbiology/Immunology, Carver College of Medicine, University of Iowa, Iowa City, Iowa, USA; University of Texas Health Science Center

**Keywords:** Candida albicans, inflammasome, pyroptosis

## Abstract

Phagocytic cells such as macrophages play an important role in the host defense mechanisms mounted in response to the common human fungal pathogen Candida albicans. *In vitro*, C. albicans triggers macrophage NLRP3-*Casp1/11*-mediated pyroptosis, an inflammatory programmed cell death pathway. Here, we provide evidence that *Casp1/11*-dependent pyroptosis occurs in the kidney of infected mice during the early stages of infection. We have also used a genome-wide screen of nonessential Σ1278b Saccharomyces cerevisiae genes to identify genes required for yeast-triggered macrophage pyroptosis. The set of genes identified by this screen was enriched for those with functions in lipid and sterol homeostasis and trafficking. These observations led us to discover that cell surface localization and/or total levels of ergosterol correlate with the ability of S. cerevisiae, C. albicans, and Cryptococcus neoformans to trigger pyroptosis. Since the mammalian sterol cholesterol triggers NLRP3-mediated pyroptosis, we hypothesized that ergosterol may also do so. Consistent with that hypothesis, ergosterol-containing liposomes but not ergosterol-free liposomes induce pyroptosis. Cell wall mannoproteins directly bind ergosterol, and we found that Dan1, an ergosterol receptor mannoprotein, as well as specific mannosyltransferases, is required for pyroptosis, suggesting that cell wall-associated ergosterol may mediate the process. Taken together, these data indicate that ergosterol, like mammalian cholesterol, plays a direct role in yeast-mediated pyroptosis.

## IMPORTANCE

Innate immune cells such as macrophages are key components of the host response to the human fungal pathogen Candida albicans. Macrophages undergo pyroptosis, an inflammatory, programmed cell death, in response to some species of pathogenic yeast. Prior to the work described in this report, yeast-triggered pyroptosis has been observed only *in vitro*; here, we show that pyroptosis occurs in the initial stages of murine kidney infection, suggesting that it plays an important role in the initial response of the innate immune system to invasive yeast infection. We also show that a key component of the fungal plasma membrane, ergosterol, directly triggers pyroptosis. Ergosterol is also present in the fungal cell wall, most likely associated with mannoproteins, and is increased in hyphal cells compared to yeast cells. Our data indicate that specific mannoproteins are required for pyroptosis. This is consistent with a potential mechanism whereby ergosterol present in the outer mannoprotein layer of the cell wall is accessible to the macrophage-mediated process. Taken together, our data provide the first evidence that ergosterol plays a direct role in the host-pathogen interactions of fungi.

## INTRODUCTION

Candida albicans is one of the most common and important human fungal pathogens (1). It is a component of the normal human microbiome with its best-characterized niches being the oral cavity and gastrointestinal tract. C. albicans causes disease in people with intact immune systems as well as those with both primary and acquired immunodeficiencies. Typically, mucosa-associated diseases such as vulvovaginal candidiasis and oral thrush affect immunocompetent patients. Patients with altered T-cell levels or function develop more severe mucosal diseases such as esophagitis. In contrast, disseminated candidiasis involving deep organs is rarely seen in patients with altered cell-mediated or humoral immunity. Instead, disseminated candidiasis is most commonly a complication of alterations of innate immune function such as those that accompany neutropenia following treatment with cytotoxic chemotherapy (2). These features of candidiasis serve to highlight the crucial role that host-C. albicans interactions play in determining the type and severity of disease that develops in at-risk patients (3). Characterizing these interactions at a fundamental level is crucial to developing a deeper understanding of pathogenesis. In turn, these insights will hopefully lead to improved care of patients through more precise risk-stratification, better diagnostic approaches, and more effective therapies.

Phagocytic cells such as neutrophils, monocytes/macrophages, and dendritic cells are critically important to the ability of the host to prevent dissemination of commensal C. albicans to deep organs (4). In response to many pathogens, or other inflammatory stimuli, macrophages are induced to assemble a multiprotein complex called the canonical inflammasome (5). Inflammasomes are composed of sensor molecules such as absent in melanoma 2 (AIM2); Nod-like receptor (NLR) CARD domain-containing protein 4 (Nlrc4); Nacht, LRR, and pyrin domain-containing protein 3 (NLRP1, -3, or -6); the adapter protein apoptosis-related speck-like protein (ASC); and caspase 1 (6). Upon stimulation, the components oligomerize into the inflammasome complex, which, in turn, serves as a platform for the activation of caspase 1. Activated caspase 1 processes cytokines such as interleukin-1β (IL-1β) and IL-18. In addition, under some conditions, inflammasomes initiate the programmed cell death pathway called pyroptosis through activation of gasdermin D, a membrane pore-forming protein. The resulting pores mediate cell lysis which releases additional mediators of inflammation. Pyroptosis is an inflammatory mode of programmed cell death, a feature that distinguishes it from apoptosis, which is noninflammatory (7). The Nlrc4 (8) and NLRP3 (9) inflammasomes are required for a normal host response to C. albicans infection. We and others have also shown that C. albicans triggers NLRP3-dependent macrophage pyroptosis and that this process contributes to the mechanism by which C. albicans kills macrophages (10, 11).

Much remains to be learned about the mechanistic details of C. albicans-induced inflammasome activation and its role in the host response to this pathogen. Here, we focus on two specific questions. First, does C. albicans trigger pyroptosis during mammalian infection? Although the components of the NLRP3 inflammasome (Nlrp3, Asc, and Casp1) are required for the normal mammalian host response to C. albicans and play a role in the processing and secretion of IL-1β and IL-18, to our knowledge, evidence of pyroptosis during mammalian infection has not been reported previously. As described below, we have observed caspase-1-dependent cell death in a murine model of disseminated candidiasis.

Second, what components of C. albicans stimulate the assembly and activation of the inflammasome? From the work of our lab and others, C. albicans triggers inflammasome assembly as it undergoes filamentation within macrophages (10, 12). Filamentation is not, however, sufficient to induce either inflammasome activation or pyroptosis since mutants such as the *upc2*ΔΔ mutant form filaments in macrophages but fail to induce pyroptosis or IL-1β secretion (10). In addition, C. albicans mutants which fail to filament in the macrophage but still trigger pyroptosis have been identified (12). O’Meara et al. also demonstrated that N-linked mannans are likely to be involved in this process (12). Moreover, purified secreted aspartyl proteases can also trigger both canonical and noncanonical inflammasome activation (13). Although specific molecules such as flagellin (Nlrc4) and double-stranded DNA (AIM2) have been shown to be cognate ligands for other inflammasome sensors, the identification of such ligands for Nlrp3 has remained elusive (6). We, therefore, were interested in attempting to further characterize yeast factors required for fungus-stimulated inflammasome activation.

To do so, we took advantage of the fact that C. albicans is not the only yeast that triggers inflammasome activation and pyroptosis. Specifically, the filamentous strain background of the model yeast Saccharomyces cerevisiae Σ1278b induces inflammasome activation while the afilamentous S288c background does not (10). This observation allowed us to take advantage of the genome-wide, nonessential gene deletion set constructed by Ryan et al. in the Σ1278b background to screen for mutants that modulate yeast-induced inflammasome activation (14). The set of genes identified by this screen was enriched genes involved in ergosterol and membrane homeostasis. Additional studies indicated that plasma membrane localization of ergosterol was required for yeast-triggered pyroptosis which, in turn, led us to discover that exposure of macrophages to ergosterol-containing liposomes leads to pyroptosis. As such, we provide the first evidence that ergosterol functions as a molecular recognition molecule in the mammalian host response to fungi.

## RESULTS

### C. albicans induces caspase-1-dependent TUNEL staining early during the infection of murine kidneys.

**FIG 1:**
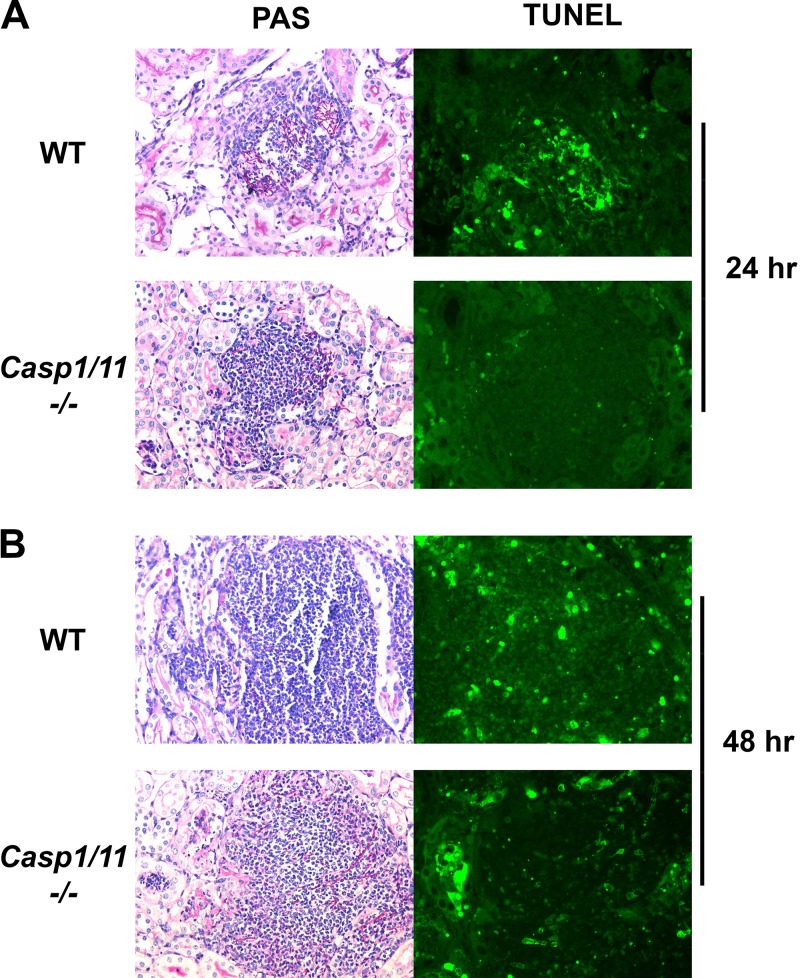
Caspase-1/11-dependent cell death occurs in the initial stages of C. albicans infection. WT and congenic *casp1/11*^−/−^ mice were infected with C. albicans SC5314 by tail vein infection. Animals were sacrificed at 24 (A) and 48 (B) h postinfection, and kidneys were harvested for histology and fungal burden. Sections from kidneys with similar fungal burdens in the two mouse backgrounds were stained with PAS stain and by TUNEL. TUNEL staining at 24 h is predominantly caspase 1/11 dependent, while caspase-1/11-independent TUNEL staining is observed at 48 h.

The components of the NLRP3 inflammasome are required for a normal host response to C. albicans in murine models of infection. Although reduced processing and secretion of IL-1β and IL-18 undoubtedly contribute to this requirement, pyroptosis is another consequence of C. albicans-triggered NLRP3 inflammasome activation (15). To our knowledge, however, evidence of pyroptosis in a mammalian model of candidiasis has not been reported previously. Both apoptosis and pyroptosis lead to DNA fragmentation, which can be observed as terminal deoxynucleotidyltransferase (TdT) dUTP nick end labeling (TUNEL)-positive cells. Pyroptosis-associated DNA fragmentation is caspase 1 dependent, while apoptosis TUNEL staining is dependent on other caspases. To determine if caspase-1-dependent cell death was occurring during disseminated candidiasis in mammals, we infected both wild-type (WT) and caspase-1-deficient mice with C. albicans strain SC5314 by tail vein injection. At 24 h and 48 h, the kidneys, which are a primary target organ of C. albicans in mice, were harvested and processed for fungal burden (CFU/gram) and histology (hematoxylin and eosin and TUNEL staining). We compared the extents of TUNEL staining in the inflammatory lesions of sections from WT and caspase-1/11-deficient mice that were matched for kidney fungal burden and overall size. As shown in Fig. 1A, wild-type lesions showed extensive TUNEL-positive cells at 24 h while caspase-1/11-deficient mice have dramatically reduced levels of TUNEL-positive cells. By 48 h postinfection, caspase-1/11-independent TUNEL staining becomes apparent, suggesting that other mechanisms of cell death contribute at this time point. Based on these data, pyroptosis appears to predominate early during infection but caspase-1-independent cell death pathways are also operative after the first 24 h of infection. This sequence of events is similar to the two-phase death kinetics displayed by C. albicans-infected macrophages *in vitro* (16): initial pyroptosis followed by nonpyroptotic cell death. As shown in Fig. 1, the lesions are heavily infiltrated with inflammatory cells, and thus, it is extremely likely that inflammatory cells contribute to the TUNEL staining of the field. Although we cannot completely rule out the possibility that renal epithelial cells contribute to the TUNEL staining of the field, no conclusive evidence of NLRP3-mediated processes has been reported for renal epithelial cells, and the majority of IL-1β/IL-18 during kidney inflammation appears to be generated by infiltrating inflammatory cells (17). Therefore, these data provide strong support for the notion that C. albicans triggers inflammatory cell pyroptosis during systemic infection.

### A genome-wide screen of nonessential genes in the Σ1278b background of S. cerevisiae for modulators of fungal inflammasome activation.

A second question with respect to C. albicans-mediated NLRP3 inflammasome activation is which pathogen molecule(s)/factor(s) triggers the process? As discussed above, the physical presence of the hyphae is not sufficient to trigger inflammasome assembly (10, 12). Previous experiments have implicated cell surface-associated mannoproteins (12), but to date, no specific ligands for the NLRP3 inflammasome have been identified (6). Therefore, we speculated that multiple factors/molecules could contribute to this process.

Although both the Cowen lab and our own lab have reported informative sub-genome-scale screens for C. albicans mutants with altered ability to trigger macrophage pyroptosis (11, 18), these approaches cannot be extended to genome-wide screens because no genome-wide, C. albicans homozygous deletion collections are currently available. As previously reported, we discovered that the filamentous strain background of S. cerevisiae, Σ1278b, is a reasonably efficient inducer of the NLRP3 inflammasome and pyroptosis in the macrophage cell line J774 (11). Although Σ1278b does not form true hyphae, it forms elongated cells within the macrophage that most closely resemble the morphology of Candida krusei (see Fig. S1 in the supplemental material), a strain that also triggers pyroptosis at levels below that observed for C. albicans (11). This is likely because carbon source starvation triggers haploid Σ1278b to undergo filamentation and, as demonstrated by Lorenz and Fink, exposure of S. cerevisiae Σ1278b to macrophages induces carbon source starvation (19). We, therefore, hypothesized that a more complete picture of the genetic requirements for NLRP3 inflammasome activation might be gained by screening the model yeast S. cerevisiae than by the use of other large collections of C. albicans deletion mutants.

10.1128/mBio.01204-18.1 FIG S1  Comparison of C. krusei and S. cerevisiae Σ1278b intramacrophage morphologies by Gram-stained coculture with J774 cells. The indicated species of yeast were cocultured with mouse J774 cells under the screening conditions described in Materials and Methods before Gram staining. The arrows indicate elongating cells of both species. Download FIG S1, TIF file, 0.3 MB. Copyright © 2018 Koselny et al. 2018 Koselny et al. This content is distributed under the terms of the Creative Commons Attribution 4.0 International license.

To that end, we screened the genome-wide collection of Σ1278b nonessential single gene deletion mutants constructed by Ryan et al. (14). Lipopolysaccharide (LPS)-primed J774 murine macrophage cells were used in order to limit the assay to second signal mediators of inflammasome activation. We used a primary screening strategy previously described for a C. albicans transcription factor deletion set screen (18). Briefly, the yeast cells were grown overnight to stationary phase, cell density was measured, and yeast cells were washed and added to the primed J774 cells at a multiplicity of infection (MOI) of 20:1. The resulting cocultures were incubated for 24 h, and the supernatants were removed. Lactate dehydrogenase (LDH) activity of the supernatants was measured and normalized to the activity of chemically lysed macrophages. Strains that induced lysis differing by 2 standard deviations (SDs) relative to wild-type reference were identified and confirmed using an independent isolate of the mutant strain. Strains with significant growth defects were identified by parallel incubation in medium without macrophages and were excluded from analysis. Finally, the mutants which were confirmed by repeat LDH assay were also examined for reduced IL-1β release. For all identified strains, the cell lysis and IL-1β release characteristics were concordant.

A total of 185 S. cerevisiae mutants with altered cell lysis/IL-1β release were identified (176 with reduced lysis and 9 with elevated lysis). This list of genes was comparable to the set of genes required for haploid invasive growth/filamentation identified by Ryan et al. (14). As expected, 99 of the genes required for macrophage lysis were also required for filamentation. Although filamentation is neither absolutely required nor sufficient for inflammasome activation (10, 12), the majority of C. albicans mutants with altered inflammasome activation are unable to filament. Thus, our data further confirm the notion that the yeast factors that trigger inflammasome activation are intimately associated with physiological responses that also accompany yeast filamentation. For example, the well-characterized regulator of S. cerevisiae filamentation, *FLO11*, is required for both LDH release and IL-1β secretion in this system. To further test the validity of our overall approach, we examined the effect of reduced expression of C. albicans orthologs of S. cerevisiae hits using the GRACE strain collection of tetracycline-promoter-regulated alleles (20). As shown in Fig. S2, there was good correlation in the strains that we tested.

10.1128/mBio.01204-18.2 FIG S2  Effect of C. albicans homologs of genes identified in S. cerevisiae screen on pyroptosis. J774 cells were exposed to C. albicans strains from the GRACE collection of tetracycline (*TET*)-regulated alleles in the presence or absence of doxycycline to shut off the expression of the indicated gene. The cocultures were processed as described in Materials and Methods for LDH release. Bars indicate means, and error bars are standard deviations of two for three biological replicates with triplicate technical replicates. Download FIG S2, TIF file, 0.03 MB. Copyright © 2018 Koselny et al. 2018 Koselny et al. This content is distributed under the terms of the Creative Commons Attribution 4.0 International license.

Removal of the genes with a known role in invasive growth left a total of 82 (77 with reduced pyroptosis and 5 with increased pyroptosis) genes with potential roles independent of filamentation (Table S1). Gene ontology (GO) term analysis of this set revealed that only genes involved in pH regulation showed significant enrichment. Vylkova and Lorenz have shown that C. albicans genes such as *STP2* are required for both extracellular alkalinization and macrophage inflammasome activation (21). However, the pH-associated C. albicans mutants identified by this group also fail to filament in macrophages. Closer inspection of the set of pH-related genes revealed six genes specifically involved in the assembly and function of the vacuolar ATPase: *VMA2/6/7/8/11/22*. Three additional genes with direct roles in vacuolar function were also identified: *VAM7*, *VPS4*, and *VAC8*. A large number of other genes have effects on vacuolar function. Therefore, we compared our set of genes with those known to have vacuolar morphology defects using the SGD database; 45/82 (55%) mutants with decreased inflammasome activation also have alterations in vacuolar function. Interestingly, 3 out of the 5 mutants that triggered increased pyroptosis are involved in endoplasmic reticulum (ER) protein trafficking (*MNL1*, *GGA2*, and *KEX2*). Overall, these data indicate that proper vacuolar function is required for S. cerevisiae to trigger pyroptosis in macrophages.

10.1128/mBio.01204-18.3 TABLE S1  S. cerevisiae deletion mutants with altered pyroptosis but normal filamentation. The gene name is shown along with whether it is involved in lipid droplet morphology or proteome (based on SGD database and references 22 to 24), contains a Upc2 consensus binding motif in the promoter region within 1 kb of the start codon (based on reference 25), displays decreased susceptibility to polyene antifungal drugs (based on reference 29), has vacuole morphology defects (SGD database), and has at least one of these composite phenotypes. The latter is noted only if the composite phenotype is absent. Gene names in bold indicate deletion mutants that induced increased levels of pyroptosis; deletion mutations in all other listed genes led to reduced levels of pyroptosis. Download TABLE S1, XLSX file, 0.01 MB. Copyright © 2018 Koselny et al. 2018 Koselny et al. This content is distributed under the terms of the Creative Commons Attribution 4.0 International license.

A systematic analysis of our set of genes led to two additional shared phenotypes. First, we found that 18/86 (20%) mutants also affected lipid droplet morphology or lipid droplet homeostasis (22); this represents a significant enrichment (*P* < 0.0001, Fisher’s exact test). Lipid droplets (LDs) are intracellular structures that contain esterified sterols and triacylglycerols involved in storage and, in conjunction with the vacuole, recycling of neutral lipids. In addition to lipids, LDs also contain a number of proteins that are involved in their formation and trafficking (23). Six of the deletions in our set of mutants are also components of the LD proteome (*ADE1*, *ADE5,7*, *ARF1*, *RPSOA*, *SAH1*, and *VMA2*). Taken together, these data indicate that 24 of the 82 genes identified as being required for S. cerevisiae-induced inflammasome activation, or over one-quarter, have a connection to LDs. Under nutrient-deprived conditions, LD-localized sterol esters (ergosterol and upstream intermediates) are mobilized as a source of membrane sterols (24). Since phagocytosed S. cerevisiae and C. albicans show characteristics of nutrient deprivation (19), these data are consistent with the notion that maintaining lipid homeostasis is required for normal pathogen function within the phagosome.

**FIG 2:**
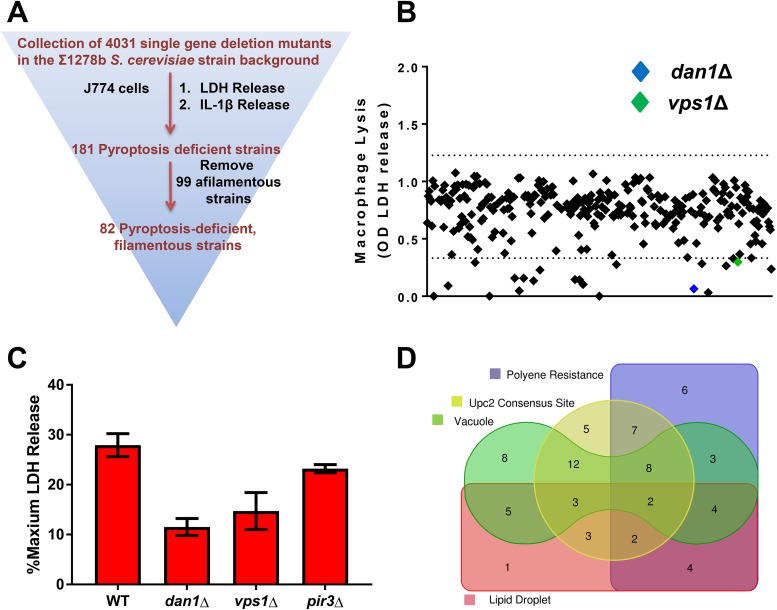
Genome-wide screen of S. cerevisiae Σ1278 background nonessential deletion mutant collection for pyroptosis-deficient filamentous strains. (A) Screening strategy and funnel. (B) Representative raw data with upper and lower cutoffs (±2 SDs) depicted as dashed lines. Two illustrative hits are indicated by colored diamonds. (C) Representative single strain confirmation of decreased LDH release from J774 murine macrophages. Percent LDH release is relative to chemically lysed cells. Bars indicate means, and error bars are standard deviations for two to three biological replicates with triplicate technical replicates. (D) Venn diagram of gene set members with roles in polyene resistance, effects on vacuole morphology, functions in lipid droplet homeostasis, and consensus motifs for Upc2, a transcription factor involved in ergosterol biosynthesis and hypoxic response.

One of the most important lipids in yeast is the sterol ergosterol. Previously, we found that the key transcriptional regulator of ergosterol biosynthesis, Upc2, was required for C. albicans-induced pyroptosis (11, 18). As a result of genome duplication, S. cerevisiae Upc2 has a paralog, Ecm22. The overlapping functions of Upc2 and Ecm22 are likely the reason that neither gene was isolated in the screen. However, *DAN1* is a cell wall mannoprotein regulated by Upc2 in S. cerevisiae (25) and showed a profound reduction in pyroptosis (Fig. 2C). In addition to *DAN1*, two other verified targets of Upc2 were identified in our screen: *HEM14* and *SAH1* (25). Previous work has shown that the motif CGTTT is part of the core consensus sequence for Upc2 binding (25). We, therefore, searched the 5′ untranslated regions (within 1 kb of the ATG) of our set of pyroptosis-associated genes for this motif. Consistent with our hypothesis, nearly one-half (42/82, 51%) of the genes in the set contain the Upc2 core motif in their promoter region. This strongly suggests that maintenance of ergosterol/sterol/lipid homeostasis is crucial for the ability of S. cerevisiae to induce pyroptosis.

Loss of Upc2 function in C. albicans leads to a reduced amount of cellular ergosterol (26). Strains with decreased ergosterol levels show reduced susceptibility to polyene antifungal drugs such as amphotericin B and nystatin (27). Polyenes bind to ergosterol, and therefore, reduced cell surface ergosterol prevents binding of the drug to the cell (28). We, therefore, wondered if the set of pyroptosis-deficient mutants was enriched for those with altered susceptibility to polyenes. Parsons et al. screened the S. cerevisiae deletion against both nystatin and amphotericin B as part of their genome-wide chemical genetic analysis (29). Based on their data, over 40% (36/82, 44%) of the pyroptosis-deficient mutants are less susceptible to either nystatin or amphotericin B. Taken together, the set of pyroptosis-deficient mutants contains a large number of mutants affecting genes involved in ergosterol or lipid homeostasis. Indeed, 78/82 genes identified in the screen had one of four interrelated characteristics: vacuole morphology defects, Upc2 binding motifs, lipid droplet connections, or reduced susceptibility to polyenes.

Based on previous experiments and prevailing models, we thought that cell wall-related proteins might represent a significant fraction of our hits (12). Instead, only two cell wall-related proteins, the Upc2-regulated *O*-mannoprotein Dan1 (30) and the mannosyltransferase Mnn5 were identified. Dan1 functions in the sterol uptake pathway as a receptor that binds extracellular sterols and facilitates their transport by Aus1 and Pdr11 (30). Consequently, its function is intimately related to sterol homeostasis. Mnn5 extends the outer chain mannans in S. cerevisiae (31) and is likely required for proper processing of Dan1. Taken together, our genetic screen indicates that genes involved in sterol homeostasis are very important for the ability of yeast to trigger pyroptosis. Since we eliminated genes involved in filamentous growth, our data also suggest that the role of ergosterol is not limited to supporting filamentous growth.

### Yeast-induced pyroptosis is dependent on ergosterol homeostasis and localization.

**FIG 3:**
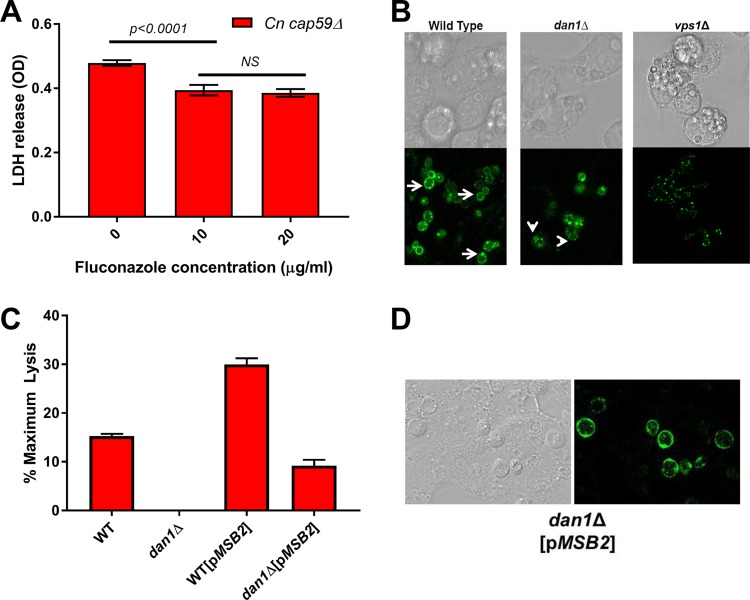
Disruption of yeast ergosterol homeostasis decreases pyroptosis. (A) Cryptococcus neoformans *cap59*Δ acapsular mutant was treated with the indicated concentrations of fluconazole overnight in YPD. The yeast cells were harvested and cocultured with J774 cells for 24 h prior to assay for cell lysis using LDH release. The bars indicate means, and error bars indicate standard deviations for two to three independent experiments performed in triplicate. Treated and untreated groups were analyzed using ordinary one-way ANOVA. *P* < 0.05 was considered statistically significant. NS, not significant. (B) WT, *dan1*Δ, and *vps1*Δ S. cerevisiae strains harboring plasmids expressing the sterol-binding domain–GFP fusion protein were exposed to J774 cells. Nonphagocytosed cells were removed by washing, and the macrophages were imaged by confocal microscopy using both DIC optics and fluorescence channels. Arrows indicate the circumferential localization of the reporter to the plasma membrane in WT cells, while arrowheads indicate exclusively cytoplasmic localization for the *dan1*Δ mutant. (C) WT and *dan1*Δ cells containing either empty vector or *MSB2* expressed from a 2μ plasmid were exposed to macrophages. Percent LDH release is relative to chemically lysed cells. Bars indicate means, and error bars are standard deviations for two to three biological replicates with triplicate technical replicates. (D) The *dan1*Δ strain containing both the ergosterol reporter and p*MSB2* was phagocytosed by J774 macrophages and analyzed as described for panel A.

The apparent dependence of yeast-triggered pyroptosis on genes involved in membrane and ergosterol homeostasis could be due to either reduced total ergosterol content or altered ergosterol distribution to the plasma membrane. In yeasts such as S. cerevisiae or C. albicans, loss-of-function mutations in ergosterol biosynthetic genes lead to decreased growth and/or prevent filamentation. Similarly, treatment with the ergosterol biosynthesis inhibitor fluconazole, although not fungicidal, blocks filamentation. O’Meara et al. demonstrated that strains deficient in ergosterol biosynthesis genes have reduced pyroptosis, but it was not possible to separate this effect from a reduction in filamentation (12). Cryptococcus neoformans mutants such as *cap59*Δ strains lacking the polysaccharide capsule trigger NLRP3-dependent pyroptosis (32). Because C. neoformans does not form filaments in macrophages, it seemed a good system to examine the effect of fluconazole on pyroptosis without the complication of reduced filamentation. Consistent with this hypothesis, *cap59*Δ cells exposed to fluconazole overnight and then cocultured with J774 cells triggered less cell lysis than untreated cells (Fig. 3A). Although the magnitude of the change in pyroptosis was modest, the cells were exposed to fluconazole only prior to coculture with macrophages because fluconazole is not active against intracellular C. neoformans. These results are consistent with our previous observation that C. albicans *upc2*ΔΔ strains, which have reduced total ergosterol, also trigger reduced pyroptosis while gain-of-function *UPC2* mutants show increases in both ergosterol and pyroptosis (11).

To test the possibility that altered localization of ergosterol affects pyroptosis, we took advantage of a recently developed fluorescent reporter of cellular ergosterol that is based on the sterol-binding domain from the Clostridium perfringens O theta-toxin which was fused to green fluorescent protein (GFP) (33). Expression of the construct from the *CUP1* promoter gave low but workable levels of protein and avoided the toxic effects of elevated steroid binding domain expression. The plasmid-borne reporter was introduced into WT, *dan1*Δ, and *vps1*Δ S. cerevisiae Σ1278b strains. The reporter strains were cocultured with J774 cells overnight. Confocal microscopy was used to localize the reporter within the phagocytosed yeast cells. As expected, WT cells showed uniform staining of the plasma membrane and scattered intracellular puncta that most likely represent lipid droplets (Fig. 3B). In contrast, the plasma membrane signal was significantly reduced in the pyroptosis-deficient *dan1*Δ and *vps1*Δ mutants; in the case of the *vps1*Δ mutant, the overall signal was also reduced. The ergosterol reporter was localized primarily to the cytoplasmic puncta in the pyroptosis-deficient mutants (Fig. 3B). These observations suggest that alterations in the cell surface localization of ergosterol reduce S. cerevisiae-induced pyroptosis.

**FIG 4:**
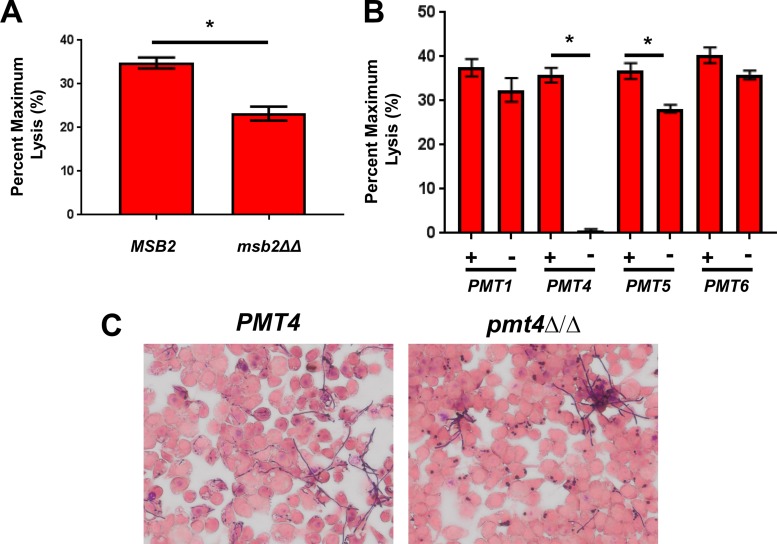
Specific *O*-mannosyltransferase isoforms are required for C. albicans-induced pyroptosis. (A and B) J774 cells were exposed to C. albicans *MSB2* and *msb2*ΔΔ strains (A) and the indicated mannosyltransferase *PMT* mutants (B) before being processed for LDH release. The bars for both panels indicate means, and error bars are the standard deviations. Asterisks indicate statistically significant differences between strains by two-sided, unpaired Student’s *t* test (A) or one-way ANOVA (B) with the limit of significance set at *P* < 0.05. (C) Gram-stained images of *PMT4* complemented strain and *pmt4*ΔΔ mutants undergoing filamentation within J774 cells.

To further explore the role of the mannoprotein Dan1 in cell surface localization of ergosterol, we overexpressed another cell surface-localized, highly *O*-glycosylated mannoprotein, the *MSB2* product (34), in both WT and *dan1*Δ cells to determine if it would affect pyroptosis. Msb2 is a member of the mucin family of proteins which participates in sterol binding in mammalian cells (35, 36) and is expressed during filamentation in both S. cerevisiae and C. albicans (37, 38). As shown in Fig. 3C, expression of *MSB2* from a 2µ multicopy plasmid increased pyroptosis in WT cells and significantly rescued pyroptosis to the *dan1*Δ strain. To determine if increased pyroptosis was accompanied by increased plasma membrane ergosterol localization, we generated strains expressing both *MSB2* and the ergosterol reporter in the *dan1*Δ strain. Indeed, plasma membrane localization was restored by overexpressing *MSB2* in the *dan1*Δ background (Fig. 4C). These data indicate that cell surface localization of ergosterol is important for the ability of yeast to trigger pyroptosis and further suggest that cell wall *O*-mannoproteins play a role in this process.

To further explore the effect of *O*-mannosylated proteins on yeast pyroptosis, we obtained a C. albicans *MSB2* homozygous deletion strain and examined its effect on pyroptosis (39). The homozygous *MSB2* deletion strain formed filaments in the macrophages but triggered reduced levels of pyroptosis (Fig. 4A). The residual pyroptosis observed in macrophages exposed to the *msb2*ΔΔ mutant suggests that other *O*-mannosylated proteins are likely to play a role in this process. If this were the case, then mutants lacking genes required for the synthesis of *O*-mannosylated proteins should also show reduced pyroptosis. To test this hypothesis, we examined a set of *O*-mannosyltransferase (Pmt) mutants for their ability to trigger pyroptosis. Of the 4 Pmt mutants that we tested (33), the *pmt4*ΔΔ mutant induced essentially no pyroptosis, while *pmt5*ΔΔ mutants showed a modest defect in pyroptosis (Fig. 4B). Previous characterization of the *pmt4*ΔΔ mutant by the Ernst lab indicates that it has very few phenotypes and grows well at temperatures up to 42°C (33). Although filamentation was somewhat delayed relative to the complemented strain, filamentous *pmt4*ΔΔ cells were present to an extent similar to the complemented strain in macrophages at the time point when LDH release was measured (Fig. 4C). Thus, the complete abrogation of pyroptosis in the *pmt4*ΔΔ mutant cannot be explained by a lack of filamentation. Msb2 shows slightly reduced SDS-PAGE mobility when expressed in the *pmt4*ΔΔ background (33), suggesting that it plays a role of *O*-mannosylation of Msb2. Furthermore, Pmt4-mediated *O*-mannosylation of Msb2 is required for full virulence of the plant pathogen Ustilago maydis (40). Consequently, our data are consistent with the notion that *O*-mannosylated proteins contribute to the activation of the inflammasome and macrophage pyroptosis by yeast.

### Ergosterol-containing liposomes trigger macrophage pyroptosis.

Cell surface localization of ergosterol could mediate yeast-triggered pyroptosis through two possible mechanisms. First, ergosterol is required for the proper trafficking and localization of cell surface proteins in yeast. Therefore, altered ergosterol localization to the plasma membrane may, in turn, disrupt the delivery and/or localization of cell surface proteins that trigger pyroptosis. In this way, ergosterol would indirectly affect the ability of yeast to trigger pyroptosis. Alternatively, ergosterol could directly trigger pyroptosis. Supporting this possibility is the fact that cholesterol, the mammalian counterpart of ergosterol, is a well-characterized trigger of the NLRP3 inflammasome (41). Although a detailed mechanism for cholesterol-induced inflammasome activation by either crystals or noncrystalline sterols has not been described, it has been extensively studied in the context of atherosclerosis. Since ergosterol is the fungal equivalent of mammalian cholesterol and is chemically similar, it seemed possible that a direct mechanism might be operative.

**FIG 5:**
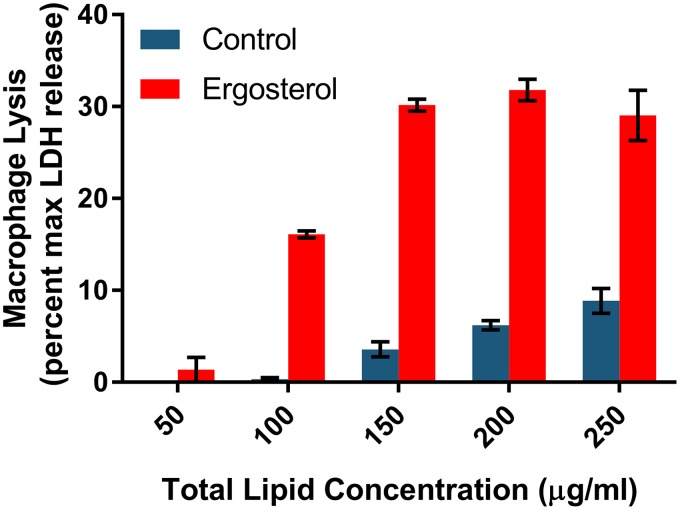
Ergosterol-containing liposomes trigger J774 cell pyroptosis. J774 cells were exposed to liposomes containing either nonsterol lipid or ergosterol. LDH release was measured and is expressed relative to chemically induced lysis. Bars indicate means, and error bars are standard deviations for two to three biological replicates with triplicate technical replicates. The differences between control and ergosterol-containing liposomes were significant (*P* < 0.05, one-way ANOVA) at all concentrations.

Recently, Zhong et al. used liposomes loaded with sterols to test the ability of noncrystalline cholesterol to trigger NLRP3 inflammasome activation (42). We, therefore, adopted this approach using ergosterol instead of cholesterol. J774 cells were exposed to commercially prepared liposomes containing either a 7:3 ratio of phosphatidylcholine (PC) to *N*-[1-(2,3-dioleoyloxy)propyl]-*N*,*N*,*N*-trimethylammonium (DOTAP) or a 4:3:3 ratio of PC to DOTAP to ergosterol. As shown in Fig. 5, macrophage pyroptosis showed a clear dose-dependent increase in pyroptosis as the concentration of ergosterol-containing lipid was increased. At concentrations where the control lipid caused essentially no LDH release, ergosterol-containing lipids trigger nearly 20% lysis. Similarly, IL-1β secretion was also stimulated by ergosterol-containing liposomes relative to liposomes lacking ergosterol (data not shown). These data are consistent with the ability of ergosterol to act as a direct activator of inflammasomes and pyroptosis in macrophages.

## DISCUSSION

Here, we have presented the first data indicating that caspase-1-dependent host cell death occurs in kidney tissue, the murine target organ for C. albicans. Specifically, we observed caspase-1-dependent host cell death at 24 h which was overtaken by caspase-1-independent death at 48 h. This pattern is consistent with *in vitro* experiments which indicate that pyroptosis dominates early during the interaction of C. albicans and macrophages but that at later time points C. albicans-mediated macrophage death occurs by other, inflammasome-independent mechanisms (10). The limitations of histological analysis prevent us from identifying exactly which host cells are undergoing caspase-1-dependent cell death. As discussed above, the majority of IL-1β secretion and NLRP3 activity associated with renal inflammation appears to be due to resident and recruited inflammatory cells. Therefore, macrophages and dendritic cells are the most likely candidates based on previous studies. For example, Lionakis et al. has shown that tissue-resident macrophages in the renal interstitium surrounding the renal tubules represent the first line of defense against the initial establishment of C. albicans infection within the kidney (43). Once this line is breached and C. albicans accesses the renal tubules, the macrophages are unable to limit infection because they cannot enter the collecting system. Thus, our observation of pyroptosis in the first 24 h of infection supports the notion that macrophage-C. albicans interactions are important during this initial phase of pathogenesis in the mammalian kidney.

The identification of specific stimuli that trigger NLRP3 assembly and activation has proven to be an elusive goal across a wide range of biological contexts. Indeed, one current model suggests that NLRP3 may respond to disrupted intracellular homeostasis rather than specific ligand-receptor-mediated interaction (44). Our genetic and biochemical data implicate ergosterol as directly contributing to the ability of filamentous yeasts such as C. albicans and S. cerevisiae to trigger the NLRP3 inflammasome. We suggest that the ability of ergosterol is mimicking cholesterol, which is a well-characterized trigger of the NLRP3 inflammasome (41, 44).

A direct role for ergosterol in triggering macrophage pyroptosis provides an explanation for previously reported observations regarding the interaction of macrophages and fungi. First, our laboratory reported that C. albicans *upc2*ΔΔ mutants, which contain less total cellular ergosterol than wild-type cells (26), trigger reduced inflammasome activation (11, 18). Conversely, C. albicans *UPC2* gain-of-function mutants have increased total cellular ergosterol (45) and increased inflammasome activation (11). Thus, if our model is correct, the effects of Upc2 mutants on pyroptosis are readily explained by alterations in the levels of overall cellular ergosterol. Second, a head-to-head comparison of the toxicity of sterols toward macrophages indicates that ergosterol is more toxic to macrophages than cholesterol (46). Although the mechanism of this toxicity was not characterized further, it is interesting to consider the fact that ergosterol does not activate liver X receptor (LXR), a key regulator of sterol efflux in liver and macrophage cells (47). Thus, we speculate that phagocytosed fungi may increase sterol concentrations within the macrophage without inducing a key compensatory response that prevents sterol toxicity and inflammasome activation.

The ability of ergosterol-containing liposomes to trigger inflammasome activation *in vitro* indicates that fungal cell surface ergosterol may be directly recognized by the macrophage. Although the majority of the cell surface ergosterol is localized to the plasma membrane and internal membrane structures and, therefore, would seem relatively inaccessible due to the presence of the outer cell wall, yeast cell walls do contain lipids, of which the majority is sterol (48). In C. albicans, isolated yeast cell walls contain 1.8% (wt/wt) lipid, which increases 2.5-fold to 4.5% in hyphae. The fraction of the cell wall that binds sterols is the mannoprotein component (49). The mannoprotein layer is the outermost region of the fungal cell wall (50). As such, the presence of ergosterol in this outer region of the cell wall provides the “opportunity” for interactions with the macrophage. The strong effects that specific mannoproteins and mannosyltransferases had on S. cerevisiae- and C. albicans-triggered pyroptosis also fit well with this model. Cell wall ergosterol has been recently implicated as a key factor in the ability of liposomal amphotericin B to traverse the fungal cell wall (51). Specifically, liposomes become trapped in the outer layers of the wall in ergosterol-deficient mutants, whereas they traffic to the plasma membrane in WT cells. Amphotericin B binds to ergosterol (28), and Walker et al. (51) suggest that this interaction occurs with cell wall ergosterol and facilitates the transport of the amphotericin B-containing liposomes through the cell wall. These observations support the notion that biologically relevant amounts of ergosterol are present within the cell wall and that cell wall ergosterol is able to interact with extracellular molecules.

Although the exact mechanism by which lipids are delivered to the cell wall is not clear, extracellular vesicles provide an attractive possibility. Extracellular vesicles contain ergosterol (52) and have been shown to be contained within the cell wall (53). Furthermore, genes required for secretory pathway trafficking affect the sterol composition of extracellular vesicles released by S. cerevisiae (54). Specifically, mutations in genes involved in Golgi transport and multivesicular body formation lead to reduced sterol content of the released extracellular vesicles. It seems likely that the membrane trafficking and lipid droplet-associated genes isolated in our screen would have similar effects on the ergosterol content of extracellular vesicles.

If cell wall-localized ergosterol plays a role in triggering pyroptosis, then it also provides an explanation for why filamentation is associated with the induction of pyroptosis. The 2.5-fold increase in ergosterol within the cell wall of hyphae relative to yeast would suggest that hyphae present more ergosterol to the macrophage than yeast cells (48). The larger overall surface area of hyphae would also be expected to further increase the “sterol load” within the macrophage relative to the compact yeast cells. Furthermore, the Konopka lab has shown that ergosterol is highly concentrated at the growing hyphal tip in C. albicans (55), which may lead to localized foci of very high sterol levels. These ideas would also explain why hypha formation in the absence of Upc2, the transcriptional activator of sterol biosynthesis genes, fails to trigger pyroptosis while hypha formation with a hyperactive allele of *UPC2* leads to increased pyroptosis.

To tie these data and literature together, we propose the following model for yeast ergosterol-mediated pyroptosis. Upon phagocytosis of the yeast, the low-nutrient environment of the phagosome induces filamentation. This leads to an increase in cell wall-associated ergosterol which may require increased ergosterol biosynthesis through the activation of Upc2, a key transcriptional regulator of sterol biosynthesis. The high concentrations of ergosterol within the cell wall of the developing hyphae may then be recognized by some as-yet-unknown receptor or may diffuse into the cytosol. The increase in cytosolic ergosterol could then mimic high levels of cholesterol and, thereby, lead to sterol toxicity and pyroptosis.

In this model, the physical presence of the hyphae is not essential *per se*. Rather, the increased amounts and/or availability of cell surface ergosterol is a key, hypha-associated factor that contributes to inflammasome activation in response to yeast. As such, our model is consistent with the work of O’Meara et al., who found that removal of mannosyl groups from the cell wall by the treatment of yeast with the mannosidase endo-β-*N*-acetylglucosaminidase H (EndoH) prevented pyroptosis (12). Removal of the ergosterol-binding mannose residues would be expected to release cell wall-associated ergosterol and, thereby, reduce their ability to trigger pyroptosis. In the case of Cryptococcus neoformans cells, it appears that the extracellular capsule blocks access to the cell wall and its associated ergosterol (32). Finally, it is also important to note that ergosterol may represent one of a number of specific stimuli that lead to fungus-mediated inflammasome activation. Regardless of the exact mechanism, these data indicate that host immune responses have developed to each of the major molecular components of the fungal cell surface, including the ergosterol component of the plasma membrane.

## MATERIALS AND METHODS

### Yeast strains and media.

The S. cerevisiae Σ1278b deletion collection was obtained from Charles Boone (14). The GRACE collection of C. albicans tetracycline-regulated strains was obtained from Merck (20). C. albicans SC5314 was obtained from the laboratory collections of C. Haidaris (Rochester). C. albicans *msb2*ΔΔ, *pmt1*ΔΔ, *pmt4*ΔΔ, *pmt5*ΔΔ, and *pmt6*ΔΔ strains were obtained from J. Ernst (37, 39). C. neoformans WT and *cap59*Δ strains were obtained from the Fungal Genetic Stock Center; the capsule-negative phenotype of the *cap59*Δ mutant was obtained experimentally prior to use. Unless otherwise stated, samples of all yeast strains were stored on agar plates and inoculated into yeast peptone-2% dextrose (YPD) or appropriate synthetic dropout liquid medium overnight at 30°C prior to use. For the animal experiment, SC5314 was passaged overnight three times prior to inoculation. Medium was prepared using standard recipes (56). S. cerevisiae strains were transformed using standard lithium acetate (LiOAc)-polyethylene glycol (PEG) methods. The tetracycline-regulated strains were incubated in YPD with doxycycline (100 µg/ml) at 30°C prior to coculture with macrophages.

### Construction of pRS316-D4H-GFP.

The D4H ergosterol sensor was made by combining the following components made by gene synthesis into the *URA3*- and centromere-containing pRS316 plasmid: the *CUP1* promoter comprised of 400 bp upstream of the *CUP1* open reading frame (ORF); an enhanced GFP, carrying F64L, S65T, and A206V mutations, recoded for the S. cerevisiae codon bias; a linker encoding GSTG; and a coding region for D4H, from the fourth domain of Clostridium perfringens O theta-toxin (residues 391 to 500; WP_070956282.1) containing an S434D mutation, recoded for the S. cerevisiae codon bias.

### Murine disseminated candidiasis model.

All mouse experimental protocols were reviewed and approved by the University of Rochester Institutional Animal Care and Use Committee. Female C57B/6 WT and *Casp1/11*^−/−^ mice (3 mice per strain) were inoculated with 200 µl of a 2.5 × 10^6^-CFU/ml suspension of C. albicans strain SC5314 by tail vein injection. Mice were euthanized at 24 and 48 h postinfection. Kidneys were dissected from each animal. One kidney from each animal was homogenized and plated to determine fungal burden. The corresponding, paired kidney was fixed in formalin and embedded in paraffin for histological analysis.

### Histological analysis of C. albicans-infected kidney tissue.

Kidneys from WT and *Casp1/11*^*−*/−^ mice were processed for periodic acid-Schiff (PAS) staining using standard methods and terminal deoxynucleotidyltransferase dUTP nick end labeling (TUNEL) using the DeadEnd fluorometric TUNEL system (Promega) according to the manufacturer’s protocol. Kidneys from WT and *Casp1/11*^*−*/−^ mice with similar fungal burdens were used to compare PAS and TUNEL staining. The PAS and TUNEL staining was performed and analyzed using sequential sections from each kidney. The images in Fig. 1 are representative of three animals per group.

### Screening of S. cerevisiae deletion mutants for altered J774 lysis and IL-1β secretion.

The working library plates from the deletion collection were thawed, and cell suspensions (10 µl) were used to inoculate a fresh deep-well 96-well plate (200 µl YPD). The plates were incubated overnight at 30°C without shaking. The yeast cells were harvested, washed with phosphate-buffered saline (PBS), and adjusted to ~2 × 10^6^ cells/ml in RPMI-1% fetal bovine serum (2×). J774 cell suspensions (100 µl of 2 × 10^5^ cells/ml) stimulated with lipopolysaccharide (LPS) (2.5 µg/ml) were combined with the yeast cell suspensions (10:1 final multiplicity of infection) and incubated at 37°C in 5% CO_2_ for 24 h. Yeast strains with severe growth defects were eliminated from screening. Control wells with no yeast were used to generate background lysis values for each plate. Supernatants (50 µl) from cocultures were removed and assayed for LDH activity using the CytoToxOne kit (Promega) according to the manufacturer’s recommendation. Wells containing J774 cells alone were treated with the kit’s lysis reagent before assay to generate the maximum level of lysis for each plate. Yeast strains that had lysis levels within ±2 standard deviations (SDs) of the plate average were identified and confirmed using individual assays. Supernatants from the confirmatory assays were also processed for IL-1β secretion using the mouse IL-1β uncoated enzyme-linked immunosorbent assay (ELISA) kit (Thermo Fisher) according to the manufacturer’s protocol.

### Confocal microscopy.

S. cerevisiae strains carrying the ergosterol reporter plasmid were cocultured with J774 cells as described above, with the exceptions that (i) the exposures were performed in 8-well chambered cover glasses instead of a 96-well plate, (ii) each well contained 2.2 × 10^5^ J774 cells, (iii) the multiplicity of infection was 5 yeast cells to 1 J774 cell, and (iv) coculture was carried out for 5 h. At the end of the coculture, cells were visualized using an inverted Leica DMi8 laser scanning confocal microscope. Transmitted light was detected using differential interference contrast (DIC). Enhanced GFP (eGFP) fluorescence was excited using a 488-nm laser and detected using a hybrid technology spectral detection unit. All images were obtained using the same microscope settings. After imaging, images were cropped and converted to TIFF files using Fiji ImageJ (57). All afterimage processing was performed identically on all of the images in Photoshop.

### Liposome-triggered J774 lysis and IL-1β secretion.

The treatment of J774 cells with liposomes was performed as previously described by Zhong et al. (42). Briefly, liposomes were purchased from Encapsula NanoSciences. The control liposome contained a 7:3 ratio of phosphatidylcholine (PC) to *N*-[1-(2,3-dioleoyloxy)propyl]-*N*,*N*,*N*-trimethylammonium (DOTAP). The ergosterol-containing liposomes were a 4:3:3 ratio of PC-DOTAP-ergosterol. J774 cells were exposed to a range of total lipid concentrations for 24 h and then processed for LDH release and IL-1β secretion as described above.
