# Supplementary figures and images for: A Genome-Wide Screen of Deletion Mutants in the Filamentous Saccharomyces cerevisiae Background Identifies Ergosterol as a Direct Trigger of Macrophage Pyroptosis
Source: mBio. 2018 Jul 31;9(4):e01204-18. doi: 10.1128/mBio.01204-18 (PMC6069111; doi:10.1128/mBio.01204-18)

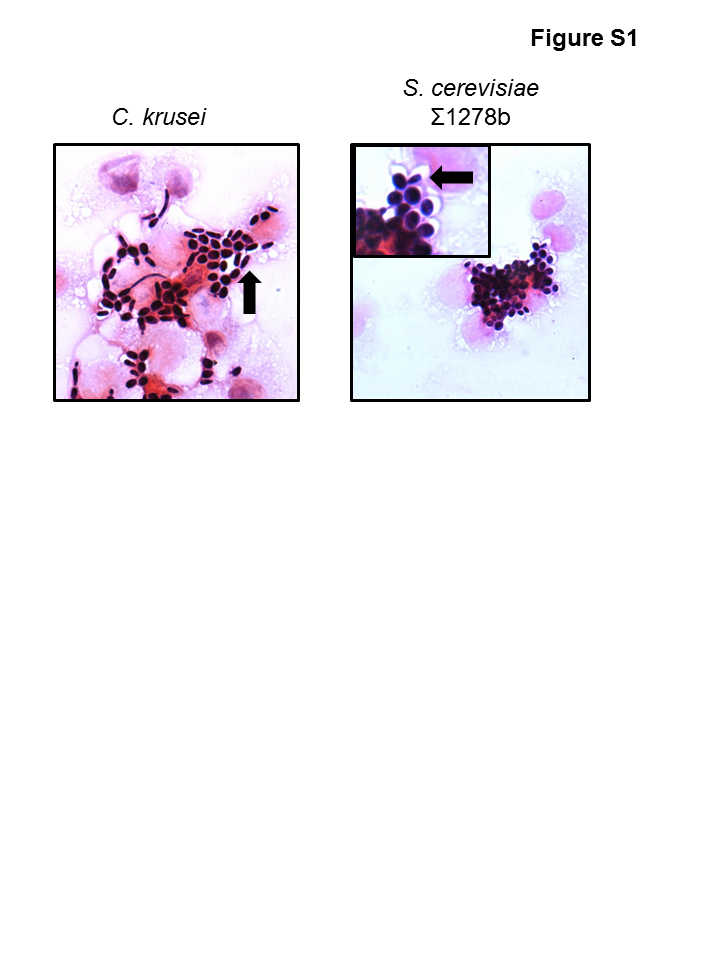

Supplement: FIG S1 [file mbo004184011sf1.tif]

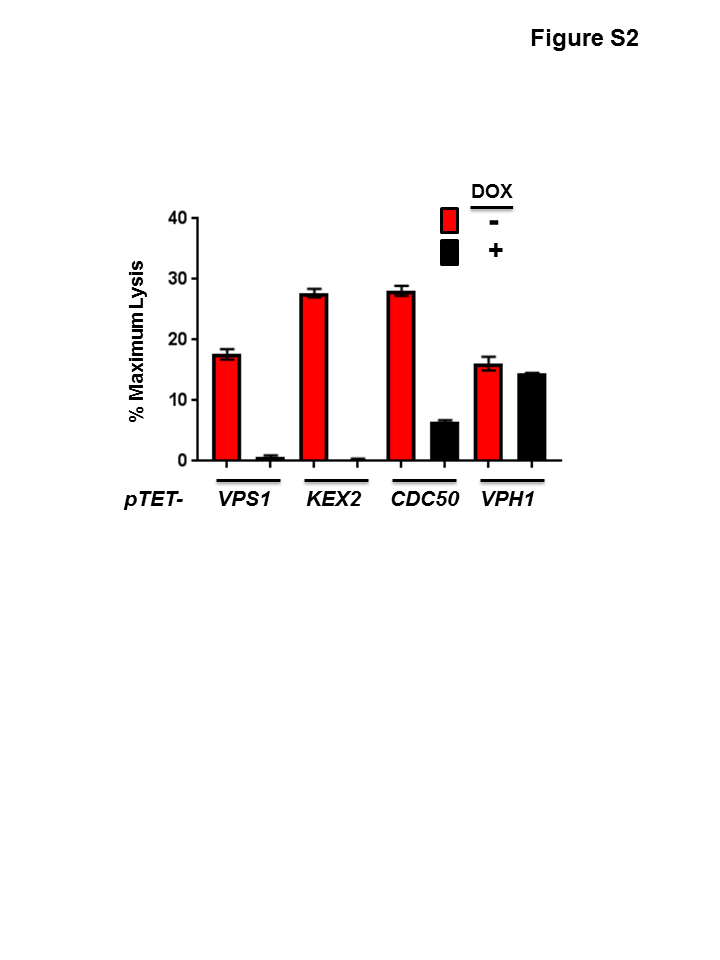

Supplement: FIG S2 [file mbo004184011sf2.tif]
